# Vaccarin Improves Myocardial Ischemia–Reperfusion Injury by Attenuating Oxidative Stress and Ferroptosis Through Reducing NOX4‐Modulated JAK2/STAT3 Pathway Activation

**DOI:** 10.1002/kjm2.70226

**Published:** 2026-04-29

**Authors:** Han Yang, Mi Zhou, Feng‐Cai Zhang

**Affiliations:** ^1^ Department of Endocrinology Nanshi Hospital of Nanyang Nanyang China; ^2^ Department of Hand Microsurgery Changde First People's Hospital Changde China; ^3^ Department of Public Health Chun'an First People's Hospital Hangzhou China

**Keywords:** ferroptosis, myocardial infarction, myocardial ischemia–reperfusion injury, oxidative stress, vaccarin

## Abstract

Vaccarin is a naturally occurring flavonoid glycoside with cardioprotective properties; however, its involvement in myocardial ischemia–reperfusion (MI/R) injury remains poorly defined. We herein investigated whether vaccarin protects cardiomyocytes from MI/R‐induced dysfunction in vivo and in vitro and examined the associated molecular mechanisms. To explore the protective effects of vaccarin, we established an MI/R injury mouse model and in vitro hypoxia/reoxygenation (H/R) injury model using H9c2 cardiomyocytes. In vivo results showed that vaccarin alleviated MI/R injury. In H9c2 cells, vaccarin partially reversed the H/R‐induced elevation of reactive oxygen species and malondialdehyde levels and restored the reduced activity of superoxide dismutase. Vaccarin also suppressed the H/R‐induced accumulation of Fe^2+^ and glutathione disulfide while restoring the levels of glutathione, solute carrier family 7 member 11, and glutathione peroxidase 4. NADPH oxidase 4 (NOX4) was subsequently identified as a putative molecular target of vaccarin. Moreover, vaccarin inhibited H/R‐induced activation of the Janus kinase 2 (JAK2)/signal transducer and activator of transcription 3 (STAT3) signaling pathway in cardiomyocytes. NOX4 overexpression counteracted vaccarin‐mediated suppression of JAK2/STAT3 signaling and blocked its antioxidative and antiferroptotic effects in H/R‐treated cardiomyocytes. Collectively, these findings indicate that vaccarin confers cardioprotection against MI/R injury by suppressing oxidative stress and ferroptosis, at least partly through inhibition of NOX4‐driven JAK2/STAT3 signaling. These results suggest that vaccarin may be a promising therapeutic candidate for the treatment of MI/R injury.

## 1 Introduction

Myocardial infarction (MI) is a life‐threatening coronary pathology associated with sudden cardiac death [1]. Currently, MI remains the most severe manifestation of coronary artery disease and is associated with high morbidity and mortality [1]. Vascular recanalization is an essential therapeutic strategy during MI management; however, restoration of blood flow can lead to myocardial ischemia–reperfusion (MI/R) injury [2]. Heart failure remains one of the most serious complications of MI/R injury [3]. Despite substantial advances in preventive strategies and therapeutic interventions for MI, post‐MI heart failure continues to be closely associated with reduced survival and poor quality of life [3]. Therefore, a comprehensive understanding of the pathological mechanisms underlying MI/R injury is essential for improving the prevention and treatment of this disease.

Multiple pathological alterations occur in myocardial tissue during MI/R injury, including cardiomyocyte loss and death [4]. Cardiomyocyte injury is closely associated with ferroptosis, a novel form of cell death characterized by disordered iron metabolism [4, 5]. In addition, ferroptosis is accompanied by several critical alterations, including impairment of antioxidant defense pathways, excessive generation of reactive oxygen species (ROS) free radicals, and lipid peroxidation [6]. Increasing evidence indicates that oxidative stress and ferroptosis play important roles in the pathogenesis of MI/R injury [7, 8]. Given their central involvement in disease progression, targeting oxidative stress and ferroptosis may provide novel therapeutic strategies for the treatment of MI/R injury.

Vaccarin (Figure 1) is a natural flavonoid glycoside isolated from *Vaccariae semen* [9]. Over the past decade, numerous studies have demonstrated that vaccarin exhibits a wide range of biological activities, including renoprotective, cardioprotective, and antidiabetic effects [10, 11, 12]. Notably, vaccarin exerts protective effects against the progression of cardiovascular diseases [11]. Moreover, a recent study showed that vaccarin alleviates renal ischemia–reperfusion (I/R) injury by suppressing inflammation and ferroptosis [13]. However, whether vaccarin exerts protective effects against MI/R injury has not yet been clarified.

**FIGURE 1:**
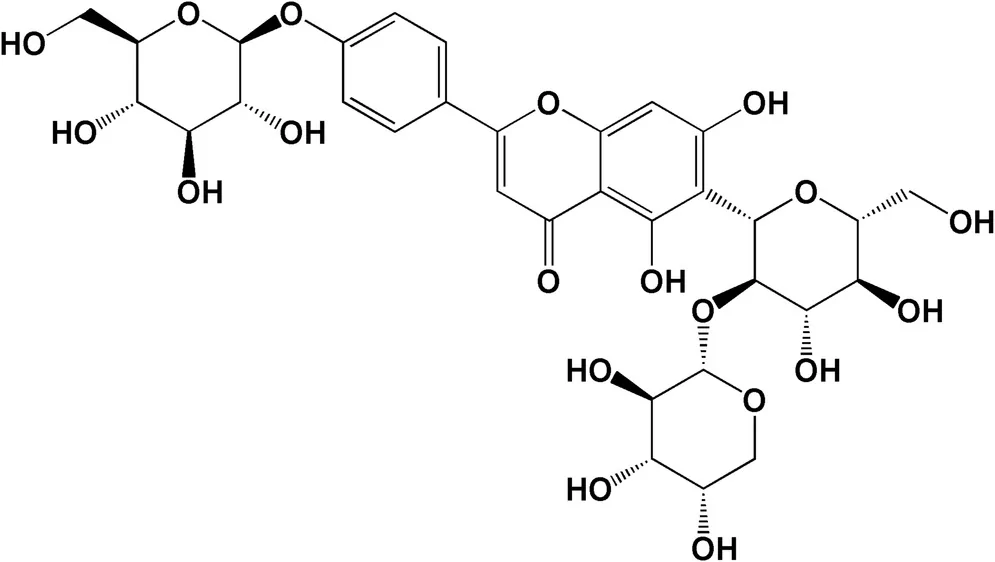
Chemical structure of vaccarin.

NADPH oxidase 4 (NOX4), a member of the NADPH oxidase family, is considered a major source of ROS production in cells [14]. Importantly, NOX4 has been identified as a molecular target of vaccarin [13]. Vaccarin alleviates cisplatin‐induced acute kidney injury by inhibiting NOX4‐derived ROS generation [10]. Increasing evidence indicates that elevated NOX4 expression is associated with MI/R‐induced oxidative stress and ferroptosis [15, 16, 17]. Based on these observations, we hypothesized that vaccarin exerts protective effects against MI/R‐induced cardiomyocyte injury by inhibiting NOX4 expression. Therefore, we herein investigated the protective role of vaccarin in cardiomyocytes subjected to MI/R‐induced dysfunction in vivo and in vitro and explored the underlying molecular mechanisms.

## 2 Materials and Methods

### 2.1 Animal Experiments

Male C57BL/6 mice (25–30 g; 10–12 weeks old) were purchased from Beijing Vital River Laboratory Animal Technology (Beijing, China). The animal experimental protocol was approved by the Animal Experimentation Ethics Committee of Nanshi Hospital of Nanyang (Approval No.: 20250105). The MI/R injury mouse model was established as previously described [18]. Briefly, left thoracotomy was conducted after anesthetizing the animals with 1.5% isoflurane. The pericardium was opened and then the left anterior descending artery (LAD) was ligated using a 6‐0 silk suture (Jinhuan Medical, Shanghai, China) passed through a PE‐10 polyethylene tube to form a reversible snare for ischemia (1 h) and reperfusion (24 h). The mice were randomly separated into four groups (*n* = 12 per group): sham, vaccarin, I/R, and I/*R* + vaccarin group. The mice in the sham group were subjected to the same procedure without LAD clamping. The mice in the vaccarin group were intraperitoneally injected with vaccarin (25 mg/kg; HY‐N1419, purity 98%, MedChemExpress, Princeton, NJ, USA) daily for 2 weeks and then subjected to the same procedure without LAD clamping. The mice in the I/R group underwent the MI/R injury procedure as described earlier. The mice in the I/R + vaccarin group were intraperitoneally injected with vaccarin (25 mg/kg) daily for 2 weeks, followed by the MI/R injury procedure. The dose of vaccarin was selected based on a previous report [10]. At the end of the experiments, blood samples were collected, and the heart tissues were removed for further assays. The heart tissues from six mice in each group were used for infarction size measurement, whereas the heart tissues from the remaining mice were used for the detection of proteins, oxidative stress indicators, and ferroptosis indicators.

### 2.2 Detection of Infarction Size

The infarction size of the hearts was detected by triphenyl tetrazolium chloride (TTC) staining [19]. Briefly, the heart tissues were removed at the end of the experiments, and then were rinsed and placed in a −80°C freezer for approximately 10 min. After slicing into five sections, the heart tissues were incubated with 2% TTC solution (Solarbio Life Sciences, Beijing, China) for 20 min in the dark, followed by immersion in 10% formalin for 24 h.

### 2.3 Measurement of Serum Creatine Kinase‐MB (CK‐MB), Cardiac Troponin I (cTnI), and Lactate Dehydrogenase (LDH)

Blood samples were collected from mice and serum was separated. The serum level of CK‐MB was determined by using a CK‐MB isoenzyme Assay Kit (H197‐1‐2; Jiancheng Bioengineering Institute, Nanjing, China). The serum level of cTnI was determined by using a cTnI Assay Kit (H149‐2‐2; Jiancheng Bioengineering Institute). The serum level of LDH was determined by using an LDH Assay Kit (A020‐2‐2; Jiancheng Bioengineering Institute, Nanjing, China) in accordance with the instructions provided by the manufacturer.

### 2.4 Cell Culture and Transfection

H9c2 cardiomyocytes (ATCC, Manassas, VA, USA) were cultured in Dulbecco's modified Eagle's medium supplemented with 10% FBS (Gibco; Carlsbad, CA, USA). An in vitro hypoxia/reoxygenation (H/R) model was established using H9c2 cells as described previously [20]. The cells were incubated in a hypoxic condition (95% N_2_ and 5% CO_2_) for 6 h and then cultured under a normoxic condition (95% air and 5% CO_2_) for 24 h. In the vaccarin treatment group, the cells were pretreated with 2.5, 5, and 10 μM vaccarin for 30 min, followed by H/R induction. To inhibit the JAK2/STAT3 pathway, JAK2 inhibitor AG490 (10 μM; HY‐12000, purity 99.86%, MedChemExpress) was used. The concentration of AG490 was selected based on a previous report [21].

To overexpress NOX4, H9c2 cells were transfected with 100 nM pcDNA3.1 plasmid carrying NOX4 (pcDNA/NOX4) or empty pcDNA3.1 plasmid (negative control) using Lipofectamine RNAiMAX reagent (Life Technologies, Grand Island, NY, USA) according to the manufacturer's instructions. Western blotting was applied to assess the transfection efficiency after 48 h of transfection.

### 2.5 CCK‐8

H9c2 cell viability was detected by CCK‐8 (Beyotime, Shanghai, China). The cells (1.5 × 10^3^ cells/well) were plated in 96‐well plates. After applying different treatments, the CCK‐8 solution was added to each well, and the plates were incubated for 1 h. The absorbance value at 450 nm was measured by using a microplate reader (Molecular Devices, Sunnyvale, CA, USA).

### 2.6 Detection of Oxidative Stress and Ferroptosis Indicators

To assess the status of oxidative stress and ferroptosis, the levels of ROS, malondialdehyde (MDA), superoxide dismutase (SOD), Fe^2+^, glutathione disulfide (GSSG), and glutathione (GSH) in the heart tissues and H9c2 cells were determined using commercial kits obtained from Beyotime or Jiancheng Bioengineering Institute, following the manufacturer's instructions.

### 2.7 Western Blotting

Western blotting was performed as described previously [18]. Primary antibodies against solute carrier family 7 member 11 (SLC7A11; ab307601; 1:1000 dilution), glutathione peroxidase 4 (GPX4; ab125066; 1:1000 dilution), Janus kinase 2 (JAK2; ab108596; 1:1000 dilution), p‐JAK2 (ab32101; 1:1000 dilution), activator of transcription 3 (STAT3; ab68153; 1:1000 dilution), p‐STAT3 (ab76315; 1:1000 dilution), and β‐actin (ab8227; 1:2000 dilution) as well as appropriate secondary antibodies (ab6721; 1:3000 dilution) were obtained from Abcam (Cambridge, MA, USA). Primary antibody against NOX4 (AF1498; 1:1000 dilution) was purchased from Beyotime. Finally, the protein bands were visualized using an enhanced chemiluminescence (ECL) kit (Beyotime). The band intensities were quantified using ImageJ software (NIH; Bethesda, MD, USA).

### 2.8 Determination of ROS

The intracellular level of ROS was detected using an ROS assay kit (S0033S; Beyotime) according to the manufacturer's instructions. After the treatments, the cells were collected and incubated with 10 μM 2,7‐dichlorodihydrofluorescein diacetate at 37°C for 20 min. The cells were then washed twice with PBS. The level of ROS was determined by a flow cytometer (BD Biosciences, San Jose, CA, USA) with an excitation/emission wavelength of 488/525 nm.

### 2.9 Bioinformatic Analysis

Targets of vaccarin were identified from SwissTargetPrediction (http://www.swisstargetprediction.ch/). The targets of myocardial ischemia were identified from GeneCards (https://www.genecards.org/). GSE160516 and GSE115568 were downloaded from Gene Expression Omnibus (GEO) datasets (https://www.ncbi.nlm.nih.gov/geo).

### 2.10 Statistical Analysis

Statistical analysis of the data was performed using GraphPad Prism version 6.0 (GraphPad Software, La Jolla, CA, USA). Data are presented as mean ± standard deviation (SD). One‐way analysis of variance (ANOVA) was adopted for multiple comparisons. *p* < 0.05 was deemed to be statistically significant.

## 3 Results

### 3.1 Vaccarin Attenuates Myocardial Injury in I/R Mice

To evaluate the protective effect of vaccarin on myocardial I/R injury in vivo, we established a mouse model of MI/R injury. Compared with the sham group, mice in the I/R group exhibited a significantly larger infarct size (Figure 2A,B). In contrast, vaccarin administration markedly reduced infarct size in I/R mice compared with untreated I/R mice (Figure 2A,B). The serum levels of CK‐MB (Figure 2C), cTnI (Figure 2D), LDH (Figure 2E), Fe^2+^ (Figure 2F), and MDA (Figure 2G) were significantly elevated in the I/R group, whereas SOD activity was reduced (Figure 2H). Vaccarin treatment partially reversed these alterations (Figure 2C–H). In addition, the protein expression levels of SLC7A11 and GPX4 were reduced in I/R mice, whereas vaccarin treatment restored their expression (Figure 2I–K). These results demonstrate that vaccarin alleviates myocardial injury in MI/R mice, and this protective effect may be associated with the modulation of oxidative stress and ferroptosis.

**FIGURE 2:**
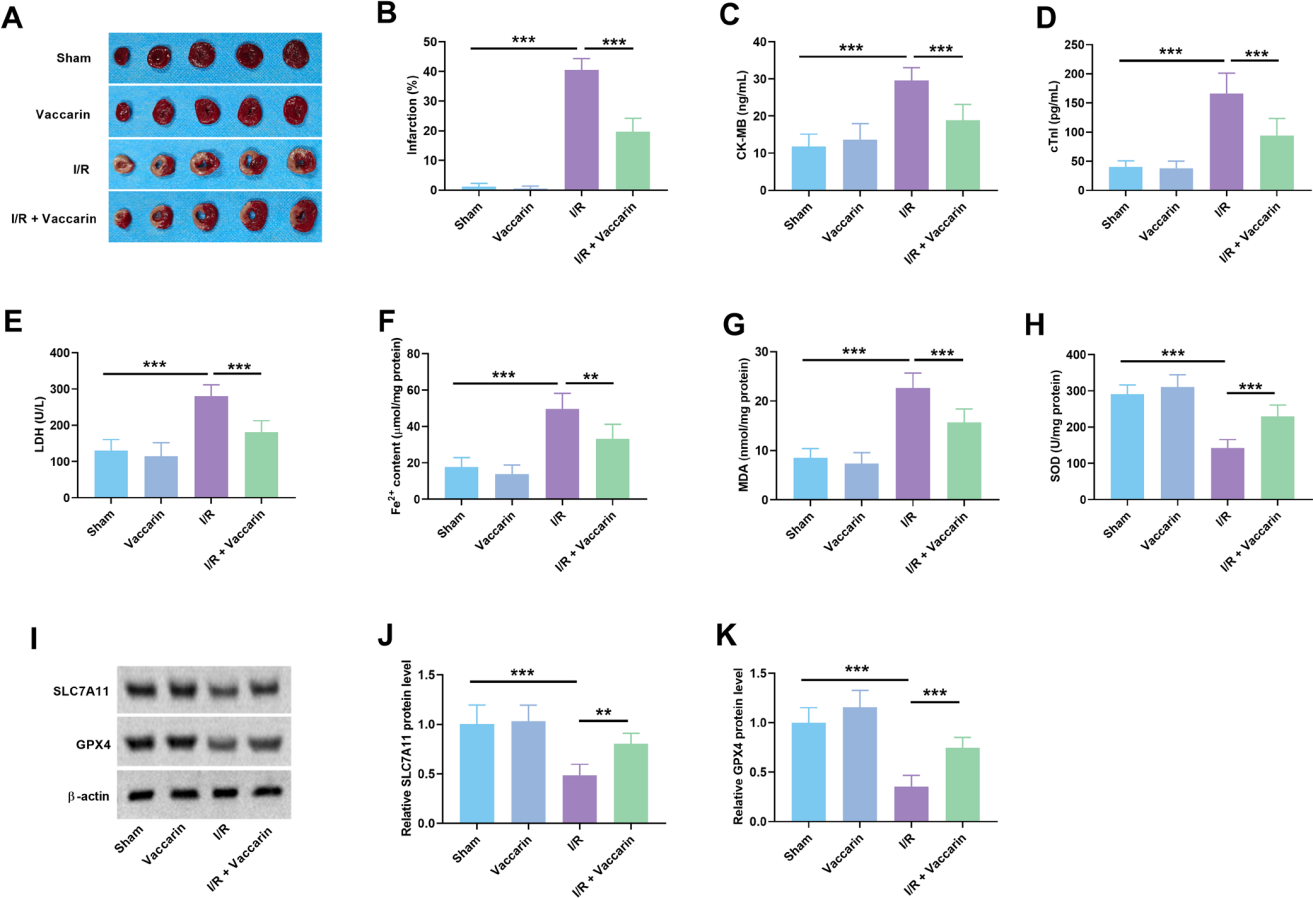
Vaccarin attenuates heart I/R injury and inhibits oxidative stress and ferroptosis in mice. (A, B) Reduced infarction size after vaccarin treatment. (C–E) Protective effects of vaccarin on myocardial injury with reduced levels of CK‐MB, cTnI, and LDH in the serum. (F–H) Changed levels of Fe^2+^, MDA, and SOD in the heart tissues. (I–K) Restored protein levels of SLC7A11 and GPX4 in the heart tissues after vaccarin treatment. Statistical analyses were performed using one‐way ANOVA, followed by Tukey's post hoc test. Data are presented as mean ± SD (*n* = 6). **p < 0.01; ***p < 0.001.

### 3.2 Vaccarin Protects Cardiomyocytes From H/R‐Induced Oxidative Stress and Ferroptosis

To further validate the protective effect of vaccarin against I/R‐induced cardiomyocyte injury and explore its role in oxidative stress and ferroptosis, an in vitro H/R injury model was established using H9c2 cardiomyocytes. Cardiomyocytes were treated with different concentrations of vaccarin (0, 2.5, 5, 10, and 20 μM) to evaluate its potential cytotoxicity. Vaccarin at concentrations of 2.5, 5, and 10 μM did not affect the cell viability of cardiomyocytes (Figure 3A). As shown in Figure 3B, the H/R‐induced reduction in cardiomyocyte viability was attenuated by vaccarin treatment (2.5, 5, or 10 μM). H/R exposure increased the production of ROS and MDA and decreased SOD activity, whereas these changes were alleviated by treatment with 2.5, 5, or 10 μM vaccarin (Figure 3C–F). Furthermore, H/R exposure increased Fe^2+^ and GSSG levels and reduced GSH levels, whereas vaccarin treatment significantly mitigated these effects (Figure 3G–I). Additionally, the protein expression levels of SLC7A11 and GPX4 were decreased in H/R‐treated cardiomyocytes, while vaccarin treatment restored their expression (Figure 3J–L). These results demonstrate that vaccarin protects cardiomyocytes from H/R‐induced oxidative stress and ferroptosis.

**FIGURE 3:**
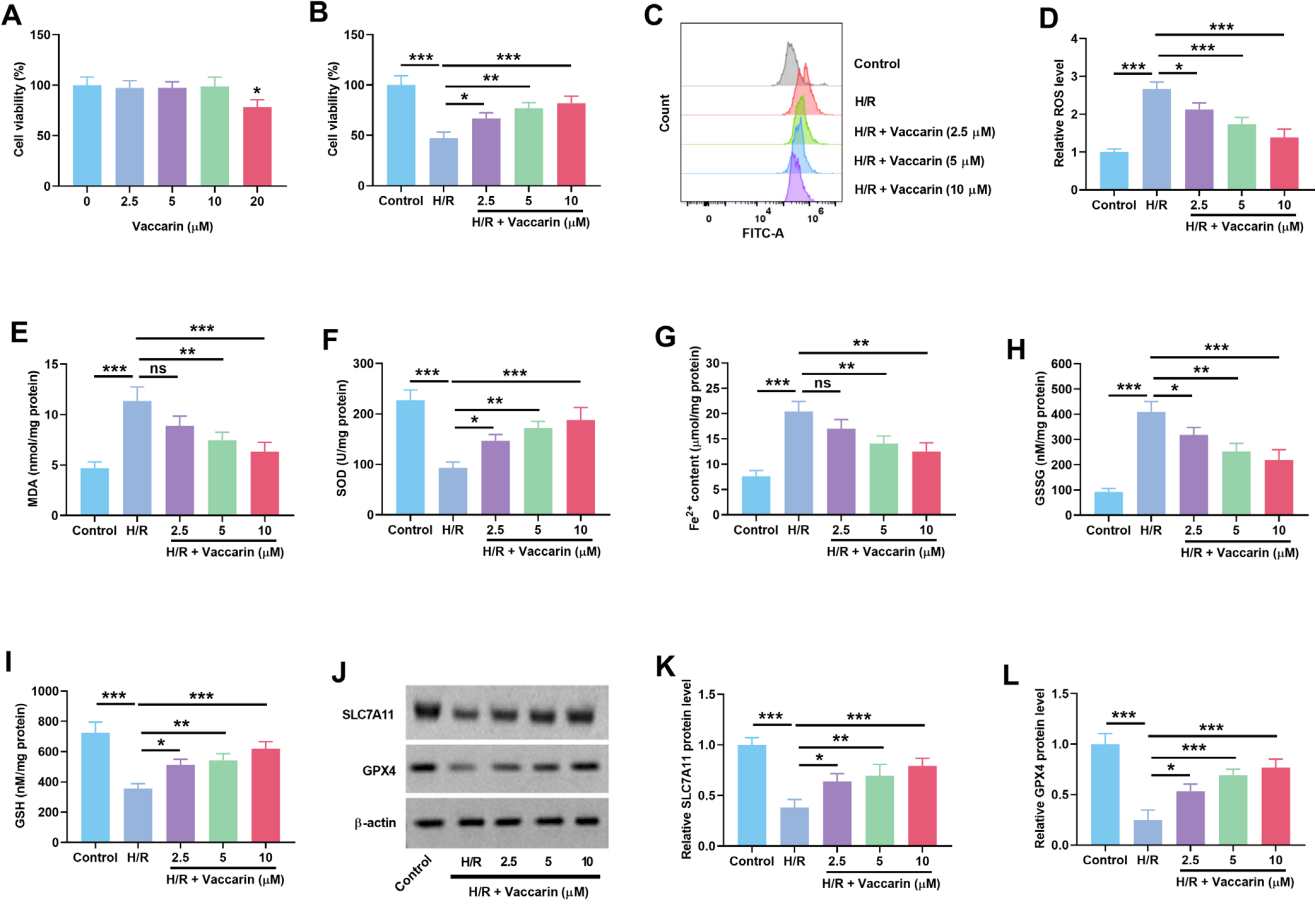
Vaccarin protects cardiomyocytes from H/R‐induced oxidative stress and ferroptosis. (A) Cell viability after treatment with different concentrations of vaccarin (0, 2.5, 5, 10, and 20 μM) for 24 h. The experiment was repeated three times in triplicate. (B) Protective effect of vaccarin (2.5, 5, and 10 μM) on cell viability in H/R‐exposed H9c2 cells. The experiment was repeated three times in triplicate. (C–F) Antioxidative effect of vaccarin in H/R‐induced H9c2 cells, as evidenced by decreased ROS and MDA production and increased SOD activity. The experiment was repeated three times in triplicate. (G–I) Reduced levels of Fe^2+^ and GSSG and increased GSH level after vaccarin treatment. The experiment was repeated three times in triplicate. (J–L) Increased protein levels of SLC7A11 and GPX4. The experiment was repeated three times. Statistical analyses were performed using one‐way ANOVA, followed by Tukey's post hoc test. Data are presented as mean ± SD. “ns” indicates no statistical significance. *p < 0.05; **p < 0.01; ***p < 0.001.

### 3.3 Vaccarin Reduces NOX4 Expression Level in Both I/R Mice and H/R‐Exposed Cardiomyocytes

To identify the potential molecular targets of vaccarin in MI/R injury, we first screened overlapping target genes associated with vaccarin and MI, and subsequently verified the expression of the key target NOX4 in vivo and in vitro. As shown in Figure 4A, 61 common target genes related to both vaccarin and MI were identified using online databases. In addition, 351 MI‐related genes were obtained from the GSE160516 and GSE115568 datasets. Finally, three overlapping genes (*HIF1A*, *PDGFRA*, and *NOX4*) were identified. Among these candidates, NOX4 has previously been reported as a molecular target of vaccarin [10]. Therefore, we further investigated the role of NOX4 in regulating the effects of vaccarin. As shown in Figure 4B, NOX4 expression was increased in I/R mice, whereas vaccarin treatment significantly reduced NOX4 expression. Consistent with the in vivo findings, H/R exposure also induced NOX4 expression in cardiomyocytes, while vaccarin treatment (2.5, 5, and 10 μM) suppressed this increase in a dose‐dependent manner (Figure 4C,D). These findings suggest that NOX4 is a key molecular target of vaccarin in MI/R injury, and that vaccarin downregulates NOX4 expression in both in vivo and in vitro models, providing a potential mechanistic basis for its cardioprotective effects.

**FIGURE 4:**
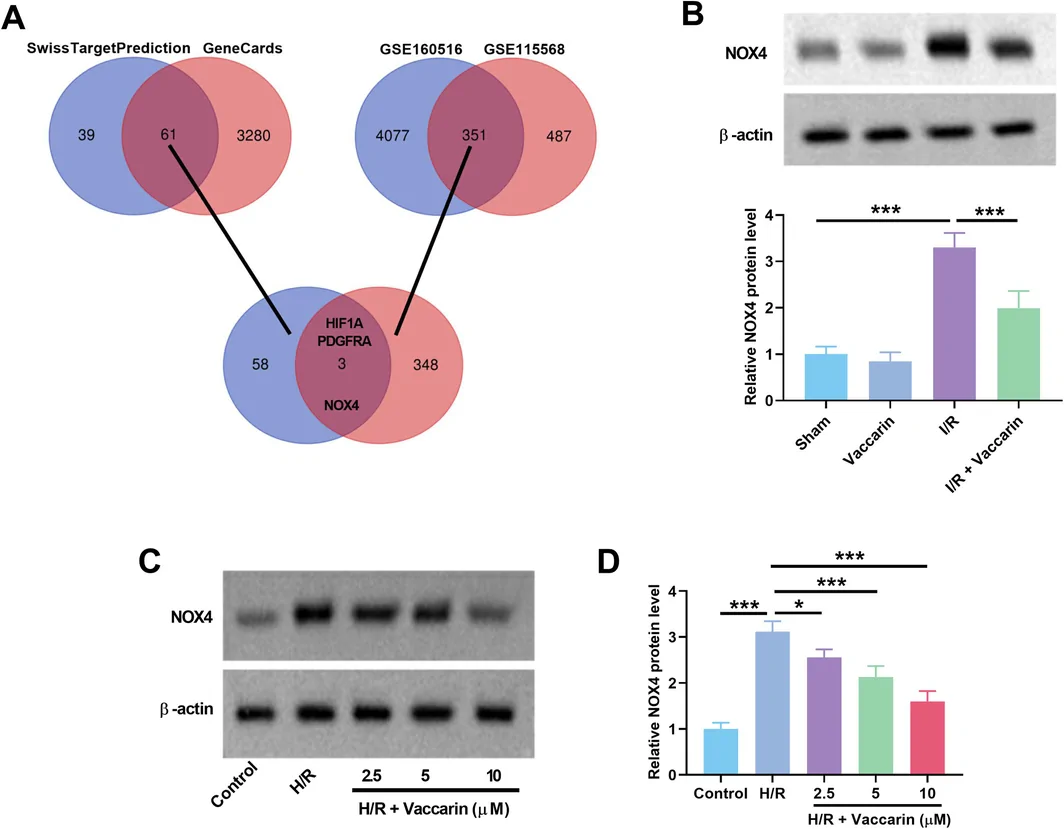
NOX4 acts as a target of vaccarin in I/R mice and H/R‐exposed cardiomyocytes. (A) Venn analysis for genes from different databases to select overlapping targets. (B) Vaccarin suppressed the NOX4 expression in I/R mice (*n* = 6). (C, D) H/R‐induced NOX4 expression in cardiomyocytes was prevented by vaccarin in a dose‐dependent manner. The experiment was repeated three times. Statistical analyses were performed using one‐way ANOVA, followed by Tukey's post hoc test. Data are presented as mean ± SD. *p < 0.05; ***p < 0.001.

### 3.4 NOX4 Mediates the Inhibitory Effect of Vaccarin on Activation of JAK2/STAT3 Signaling in H/R‐Treated Cardiomyocytes

Based on the above findings identifying NOX4 as a key target of vaccarin, we further explored the downstream signaling pathways of NOX4 to clarify the molecular mechanism underlying the protective effects of vaccarin. A previous study has shown that the NOX4‐mediated JAK/STAT signaling pathway is involved in renal I/R injury [22]. Additionally, the activation of the JAK2/STAT3 pathway has been closely associated with MI/R injury [23, 24]. Therefore, we examined the involvement of the JAK2/STAT3 pathway in the vaccarin–NOX4 regulatory axis. As shown in Figure 5A–C, the ratios of p‐JAK2/JAK2 and p‐STAT3/STAT3 were increased in I/R mice, indicating activation of JAK2/STAT3 signaling. Vaccarin treatment markedly suppressed the activation of JAK2/STAT3 signaling in I/R mice. Next, a NOX4‐overexpressing H9c2 cell line was generated. Following transfection with pcDNA/NOX4, NOX4 expression increased approximately 2.2‐fold (Figure 5D,E). Vaccarin treatment (2.5, 5, and 10 μM) reduced the H/R‐induced increases in p‐JAK2/JAK2 and p‐STAT3/STAT3 levels; however, these inhibitory effects were reversed by NOX4 overexpression (Figure 5F–H). Furthermore, treatment with AG490, a JAK2 inhibitor, attenuated the effect of NOX4 overexpression on JAK2/STAT3 signaling, suggesting that NOX4 mediates the inhibitory effect of vaccarin on this signaling pathway (Figure 5F–H). Collectively, these results demonstrate that vaccarin suppresses activation of the JAK2/STAT3 signaling pathway during MI/R injury, and that this inhibitory effect is mediated, at least in part, through downregulation of NOX4 expression.

**FIGURE 5:**
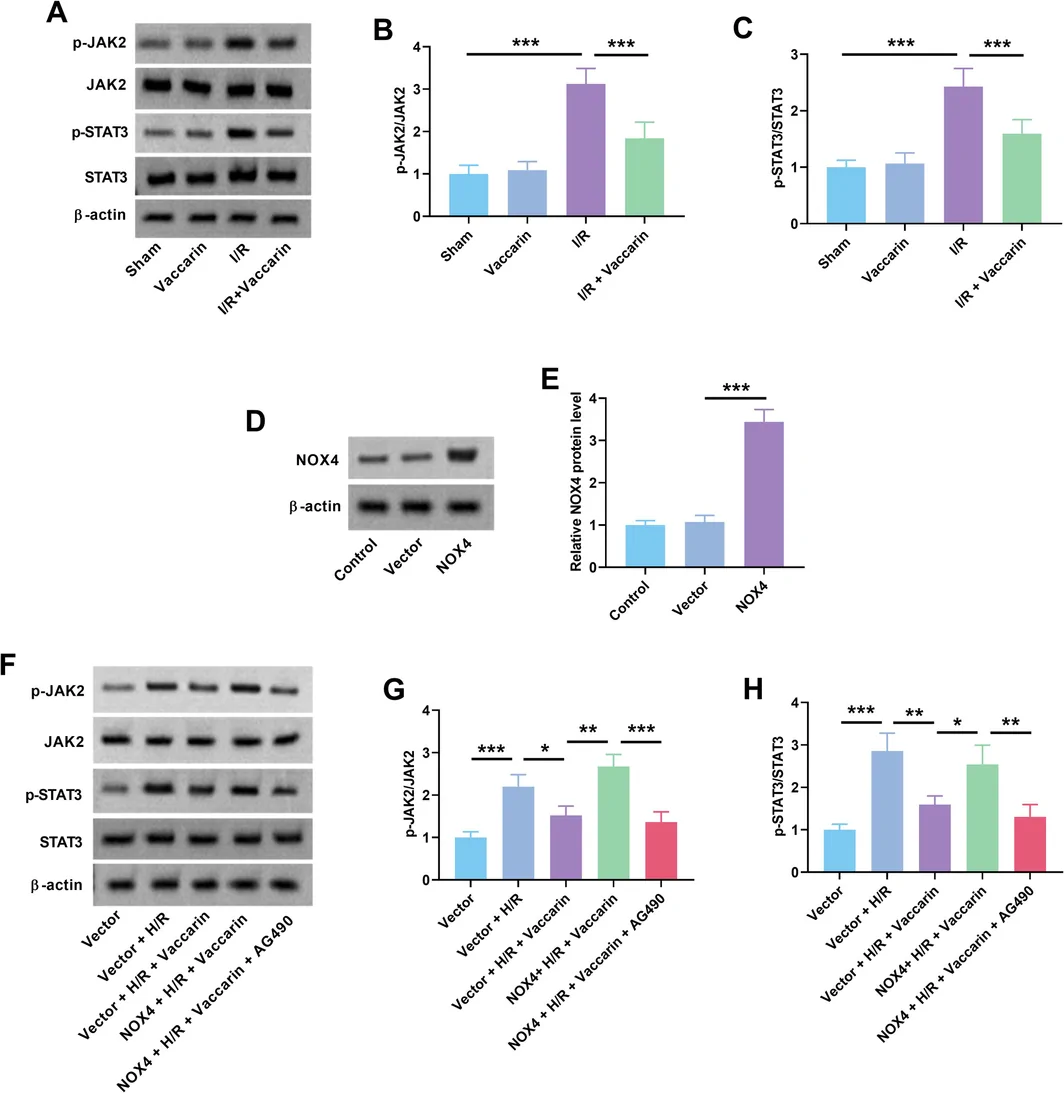
Vaccarin prevents the activation of JAK2/STAT3, which is mediated by NOX4. (A–C) Inhibitory effect of vaccarin on JAK2/STAT3 signaling in I/R mice (*n* = 6). Statistical analyses were performed using one‐way ANOVA, followed by Tukey's post hoc test. (D, E) NOX4‐overexpressing H9c2 cells were constructed through transfection with pcDNA/NOX4. The experiment was repeated three times. Statistical analyses were performed using one‐way ANOVA, followed by Dunnett's post hoc test. (F–H) H9c2 cells were transfected with an empty pcDNA3.1 plasmid (vector) or pcDNA/NOX4. After 24 h of transfection, the cells were pretreated with vaccarin (10 μM) and/or AG490 (10 μM) for 30 min, followed by H/R induction. Western blotting was conducted to determine the expression of p‐JAK2, JAK2, p‐STAT3, and STAT3. The experiment was repeated three times. Statistical analyses were performed using one‐way ANOVA, followed by Tukey's post hoc test. Data are presented as mean ± SD. *p < 0.05; **p < 0.01; ***p < 0.001.

### 3.5 NOX4/JAK2/STAT3 Signaling Mediates the Inhibitory Effects of Vaccarin on H/R‐Induced Oxidative Stress and Ferroptosis

To determine whether the NOX4/JAK2/STAT3 signaling pathway mediates the antioxidative and antiferroptosis effects of vaccarin in MI/R injury, rescue experiments were performed using NOX4 overexpression and the JAK2 inhibitor AG490. As shown in Figure 6A–D, the vaccarin‐induced decrease in ROS and MDA levels and increase in SOD activity were reversed by NOX4 overexpression. In contrast, inhibition of JAK2 with AG490 reduced ROS and MDA levels and increased SOD activity. Furthermore, vaccarin treatment attenuated the H/R‐induced increase in Fe^2+^ and GSSG levels and restored the reduced levels of GSH, SLC7A11, and GPX4. However, these effects were reversed by NOX4 overexpression (Figure 6E–J). Treatment with AG490 mitigated the effects of NOX4 overexpression on Fe^2+^, GSSG, GSH, SLC7A11, and GPX4 levels (Figure 6E–J). These findings indicate that the NOX4/JAK2/STAT3 signaling pathway mediates the suppressive effects of vaccarin on H/R‐induced oxidative stress and ferroptosis in cardiomyocytes.

**FIGURE 6:**
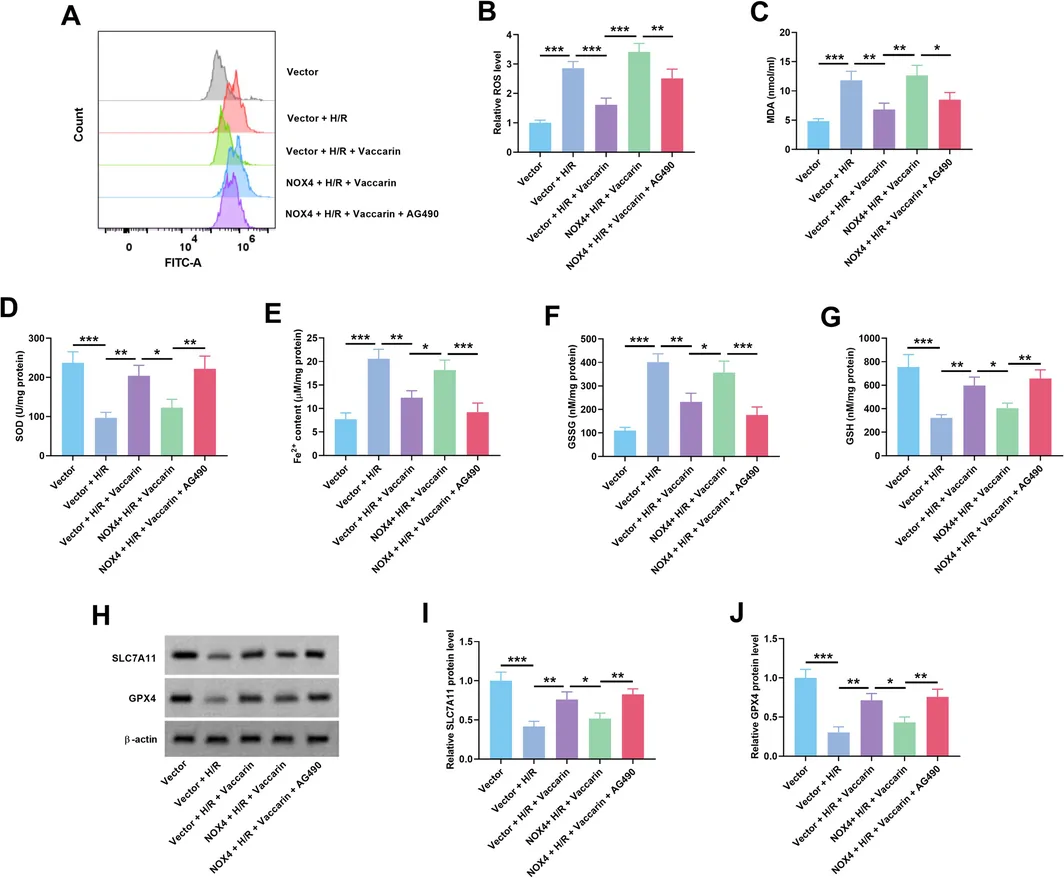
The inhibitory effects of vaccarin on H/R‐induced oxidative stress and ferroptosis in cardiomyocytes are mediated by the NOX4/JAK2/STAT3 signaling pathway. H9c2 cells were transfected with an empty pcDNA3.1 plasmid (vector) or pcDNA/NOX4. After 24 h of transfection, the cells were pretreated with vaccarin (10 μM) and/or AG490 (10 μM) for 30 min, followed by H/R induction. (A–D) ROS and MDA production and SOD activity were determined. The experiment was repeated three times in triplicate. (E–G) The levels of Fe^2+^, GSSG, and GSH were determined. The experiment was repeated three times in triplicate. (H–J) The expression levels of SLC7A11 and GPX4 were determined. The experiment was repeated three times. Statistical analyses were performed using one‐way ANOVA, followed by Tukey's post hoc test. Data are presented as mean ± SD. *p < 0.05; **p < 0.01; ***p < 0.001.

## 4 Discussion

Vaccarin is a natural flavonoid glycoside with reported cardioprotective effects [11]. Vaccarin inhibits oxidized low‐density lipoprotein‐induced inflammation, endothelial‐mesenchymal transition, cellular injury, and apoptosis in human umbilical vein endothelial cells [11]. In addition, vaccarin selectively protects vascular endothelial cells from high glucose‐induced dysfunction [12]. Furthermore, vaccarin alleviates septic cardiomyopathy by reducing cardiac oxidative stress and inflammation [25]. In the present study, we investigated the effects of vaccarin on MI/R injury and found that vaccarin attenuated myocardial injury in I/R mice and the H/R‐induced reduction in cardiomyocyte viability. These findings indicate that vaccarin exerts protective effects against I/R‐induced cardiac and cardiomyocyte injury.

Ferroptosis and oxidative stress are key contributors to MI/R injury and represent potential therapeutic targets for the prevention and treatment of this disorder [7, 8]. Several bioactive compounds exhibit therapeutic potential in MI/R injury by suppressing ferroptosis and oxidative stress. For example, ginsenoside Re attenuates cardiac dysfunction during MI/R injury by inhibiting ferroptosis [26]. Glycyrrhizin attenuates MI/R injury and improves cardiac function by suppressing ferroptosis and oxidative stress [27]. Furthermore, urolithin A ameliorates MI/R injury by attenuating oxidative stress and ferroptosis through activation of the Nrf2 signaling pathway [28]. These findings suggest that targeting ferroptosis and oxidative stress represents an effective therapeutic strategy for MI/R injury. Furthermore, vaccarin exhibits antiferroptotic and antioxidative activities in various pathological conditions. For example, in a mouse model of renal fibrosis, vaccarin reduces ROS, MDA, lipid peroxidation, and Fe^2+^ levels while increasing GSH levels, indicating its inhibitory effects on ferroptosis and oxidative stress [29]. Fan et al. reported that vaccarin alleviates renal I/R injury through its antiferroptotic and antioxidative effects [13]. Vaccarin also attenuates hydrogen peroxide‐induced oxidative stress injury in human umbilical vein endothelial cells [30]. In addition, vaccarin can prevent septic cardiomyopathy by reducing cardiac oxidative stress in both septic mice and cardiomyocytes [25]. Consistent with these findings, the present study demonstrated that vaccarin alleviates H/R‐induced oxidative stress and ferroptosis in both mice and cardiomyocytes.

The STAT family comprises several members, including STAT1‐STAT6 [31]. Accumulating evidence indicates that the STAT signaling pathway is closely associated with various pathological processes involved in cardiovascular diseases, including ischemic heart disease [32]. Studies investigating the molecular mechanisms of STAT signaling have shown that members of the STAT family are regulated by multiple upstream molecules [33]. Among these regulators, the JAK family plays a critical role in the recruitment and activation of STAT proteins [34]. The JAK/STAT signaling pathway participates in ischemic stress responses and remains a potential therapeutic target for I/R injury [34]. Notably, accumulating evidence indicates that the JAK2/STAT3 pathway plays dual roles in MI/R injury, with both activation and inhibition of this pathway reported to confer cardioprotection under different experimental conditions. It has been reported that JLX001, loganin, cucurbitacin B, and L‐theanine mitigate MI/R injury by activating the JAK/STAT pathway [23, 35, 36, 37]. Conversely, inhibition of JAK3 has been demonstrated to protect mice from MI/R injury [38]. Moreover, *Cornus officinalis* total glycosides and ganoderic acid A protect against MI/R injury partially by inhibiting activation of the JAK2/STAT3 pathway [39, 40]. In the present study, we found that treatment with vaccarin inhibited activation of the JAK2/STAT3 signaling pathway in both MI/R mice and cardiomyocytes, suggesting that modulation of the JAK2/STAT3 pathway contributes to the cardioprotective effects of vaccarin. The NOX4‐mediated JAK/STAT signaling pathway is involved in renal I/R injury [22]. For example, ellagic acid alleviates renal I/R injury by suppressing oxidative stress and apoptosis through regulation of the NOX4/JAK/STAT signaling pathway [22]. Based on these findings, we further investigated the role of NOX4 in mediating the cardioprotective effects of vaccarin. Our results showed that vaccarin reduced NOX4 expression in both I/R mice and H/R‐treated cardiomyocytes. Moreover, the inhibitory effects of vaccarin on H/R‐induced oxidative stress and ferroptosis in cardiomyocytes were mediated through the NOX4‐dependent JAK2/STAT3 signaling pathway.

In conclusion, the present study demonstrates that vaccarin exerts cardioprotective effects against MI/R injury. Vaccarin ameliorates MI/R‐induced oxidative stress and ferroptosis by suppressing the NOX4‐mediated JAK2/STAT3 signaling pathway (Figure 7). These findings suggest that vaccarin may be a promising therapeutic candidate for the treatment of MI/R injury.

**FIGURE 7:**
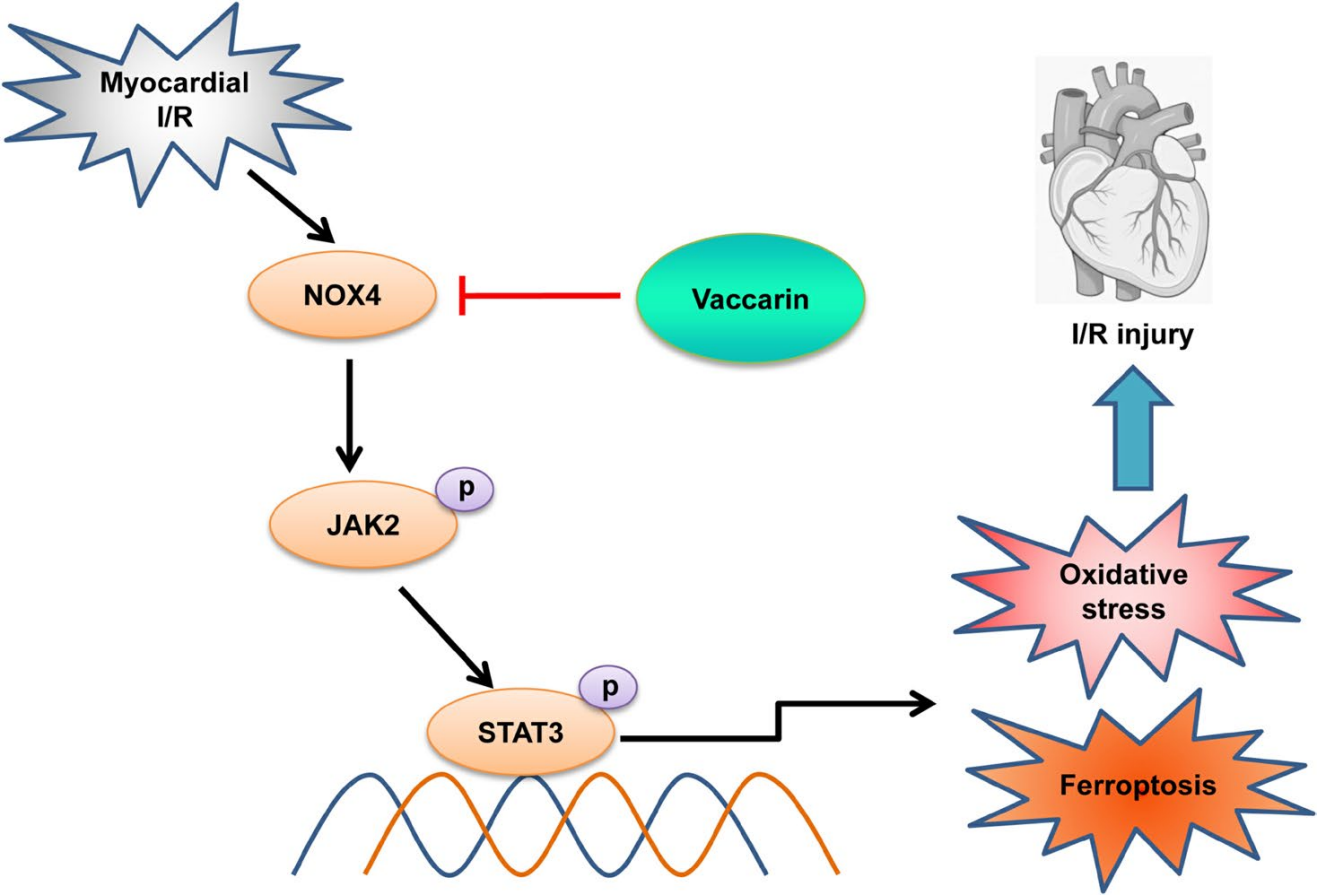
Mechanisms underlying the cardioprotective role of vaccarin in MI/R injury. Vaccarin suppressed the myocardial I/R‐induced NOX4 expression, thus inactivating the JAK2/STAT3 signaling. The suppression of NOX4‐mediated JAK2/STAT3 signaling contributed to the antioxidative and antiferroptotic effects of vaccarin in MI/R injury.

## Conflicts of Interest

The authors declare no conflicts of interest.

## Data Availability

The data that support the findings of this study are available from the corresponding author upon reasonable request.
